# Supplementary material for: Ethnicity and Clinical Outcomes in Patients Hospitalized for COVID-19 in Spain: Results from the Multicenter SEMI-COVID-19 Registry
Source: J Clin Med. 2022 Mar 31;11(7):1949. doi: 10.3390/jcm11071949 (PMC8999367; doi:10.3390/jcm11071949)
Supplement: Supplementary file 1 [file jcm-11-01949-s001.zip › jcm-1624478-supplementary.pdf]

Table S1. Full Multivariable Logistic Regression Model for In Hospital Mortality.

	Model Adjusted [a]	
Independent variables	**Odds ratio (95% CI)**	**p-value**
**Ethnicity**		
**Latino-Americans**	1.11 (0.90-1.36)	0.320
**North-Africans**	1.05 (0.70-1.60)	0.786
**Sub-Saharans**	0.28 (0.10-0.79	0.017
**Asian**	1.50 (0.77-2.93)	0.320
**European**	Ref	
**Age group, years**		
**0–44**	Ref	
**45–64**	3.07 (1.84-5.11)	<0.001
**65+**	6.99 (4.17-11.72)	<0.001
**Sex**		
**Female**	Ref	
**Male**	1.42 (1.32-1.53)	<0.001
**Wave**		
**First**	Ref	
**Second**	0.76 (0.70-0.83)	<0.001
**Nosocomial**		
**No**	Ref	
**Yes**	1.77 (1.54-22.04)	<0.001
**CCI category**		
**0-2**	Ref	
**3-4**	3.72 (3.18-4.36)	<0.001
**5+**	9.14 (7.81-10.69)	<0.001

CCI: Charlson Comorbidity Index; Ref., reference

[a] Adjusted for age group, sex, wave, place of acquisition and baseline Charlson comorbidity index group.

Table S2. Full Multivariable Logistic Regression Model for Intensive Care Unit admission

	**Model Adjusted** [a]	
Independent variables	**Odds ratio (95% CI)**	**p-value**
**Race/ethnicity**		
**Latino-Americans**	1.37 (1.17-1.60)	<0.001
**North-Africans**	1.74 (1.26-2.41)	0.001
**Sub-Saharans**	1.03 (0.57-1.86)	0.903
**Asian**	1.49 (0.87-2.56)	0.145
**European**	Ref	
**Age group, years**		
**0–44**	Ref	
**45–64**	1.62 (1.31-2.01)	<0.001
**65+**	1.47 (1.15-1.88)	0.002
**Sex**		
**Female**	Ref	
**Male**	1.86 (1.68-2.05)	<0.001
**Wave**		
**First**	Ref	
**Second**	1.09 (0.98-1.21)	0.081
**Nosocomial**		
**No**	Ref	
**Yes**	0.88 (0.68-1.12)	0.372
**CCI category**		
**0-2**	Ref	
**3-4**	1.25 (1.09-1.43)	0.001
**5+**	0.41 (0.35-4.91)	<0.001

CCI: Charlson Comorbidity Index; Ref., reference

[a] Adjusted for age group, sex, wave, place of acquisition and baseline Charlson comorbidity index group.

Table S3. Full Multivariable Logistic Regression Model for use of invasive mechanical ventilation

	**Model Adjusted** [a]	
Independent variables	**Odds ratio (95% CI)**	**p-value**
**Race/ethnicity**		
**Latino-Americans**	1.43 (1.21-1.71)	<0.001
**North-Africans**	1.50 (1.01-2.21)	0.051
**Sub-Saharans**	1.01 (0.50-2.01)	0.974
**Asian**	1.23 (0,63-2.38)	0.534
**European**	Ref.	
**Age group, years**		
**0–44**	Ref	
**45–64**	1.99 (1.53-2.59)	<0.001
**65+**	1.93 (1.44-2.58)	<0.001
**Sex**		
**Female**	Ref	
**Male**	1,81 (1,62-2.02)	<0.001
**Wave**		
**First**	Ref	
**Second**	0.78 (0.69-0.88)	<0.001
**Nosocomial**		
**No**	Ref	
**Yes**	0.79 (0.59-1.07)	0.135
**CCI category**		
**0-2**	Ref	
**3-4**	1.30 (1.12-1.51)	0.001
**5+**	0.37 (0.31-0.45)	<0.001

CCI: Charlson Comorbidity Index; Ref., reference

[a] Adjusted for age group, sex, wave, place of acquisition and baseline Charlson comorbidity index group.

Table S4. Full Multivariable Logistic Regression Model for use of combined outcome

	**Model Adjusted [a]**	
**Independent variables**	**Odds ratio (95% CI)**	**p-value**
**Race/ethnicity**		
**Latino-Americans**	1.13 (0.98-1.31)	0.082
**North-Africans**	1.27 (0.93-1.72)	0.122
**Sub-Saharans**	0.79 (0.46-1.35)	0.398
**Asian**	1.521 0.94-2.44)	0.085
**European**	Ref	
**Age group, years**		
**0–44**	Ref	
**45–64**	1.49 (1.22-1.84)	<0.001
**65+**	2.34 (1.88-2.91)	<0.001
**Sex**		
**Female**	Ref	
**Male**	1,51 (1,42-1.61)	<0.001
**Wave**		
**First**	Ref	
**Second**	0.78 (0.69-0.91)	<0.001
**Nosocomial**		
**No**	Ref	
**Yes**	1.56 (1.36-1.78)	<0.001
**CCI category**		
**0-2**	Ref	
**3-4**	1.86 (1.67-2.08)	<0.001
**5+**	3.47 (3.10-3.88)	<0.001

CCI: Charlson Comorbidity Index; Ref., reference

[a] Adjusted for age group, sex, wave, place of acquisition and baseline Charlson comorbidity index group.

Table S5. Outcomes of Patients with Confirmed COVID-19 by Ethnic Group during first wave

	**M**	**Total (n=16879)**[ϒ]	**Latin Americans (n=1375; 8.1%)**[ϒ]	**North Africans (n=163; 1.0%)**[ϒ]	**Sub-Saharan Africans (n=73; 0.4%)**[ϒ]	**Asians (n=76; 0.5%)**[ϒ]	**Europeans (n=1519; 90.0%)**[ϒ]	**Global *p***	**LA vs. E *p***	**NA vs. E *p***	**SS vs E *p***	**Asian vs. E *p***
**Main outcomes, n (%)**												
**In-hospital mortality**	0	3519 (20.8)	107 (7.9)	13 (8.0)	3 (4.1)	8 (10.5)	3388 (22.3)	<0.001	<0.001	<0.001	<0.001	0.014
**Intensive care unit admission**	0	1577 (9.3)	170 (12.4)	20 (12.3)	9 (12.3)	10 (13.2)	1368 (9.0)	<0.001	<0.001	0.141	0.324	0.208
**Invasive mechanical ventilation**	80	1292 (7.7)	148 (10.8)	14 (8.7)	8 (11.1)	7 (9.2)	1115 (7.4)	<0.001	<0.001	0.524	0.227	0.541
**Composite outcome**	50	4484 (26.6)	216 (15.8)	28 (17.5)	12 (16.7)	17 (22.4)	4211 (27.8)	<0.001	<0.001	0.004	0.035	0.292
**Other outcomes, n (%)**												
**Non-invasive mechanical ventilation**	85	934 (5.6)	84 (6.1)	7 (4.3)	7 (9.7)	3 (3.9)	833 (5.5)	0.388	0.334	0.520	0.119	0.551
**High-flow oxygen therapy**	145	1438 (8.6)	137 (10.0)	18 (11.3)	9 (12.5)	7 (9.2)	1267 (8.4)	0.126	0.040	0.189	0.202	0.802
**Length of hospital stay, days, median (IQR)**	134	9 (5-14)	8 (6-14)	10 (7-17)	12 (7-17)	9 (6-15)	9 (5-14)	0.004	0.003	0.003	<0.001	0.235
**Length of hospital stay >10 days, n (%)**	134	7553 (44.9)	596 (43.4)	86 (53.1)	44 (60.3)	35 (46.1)	6792 (44.9)	0.013	0.524	0.013	0.012	0.632

[ϒ] First wave; M: Missing value; E: Europeans; LA: Latin Americans; NA: North Africans; SS: Sub-Saharan Africans

Table S6. Outcomes of Patients with Confirmed COVID-19 by Ethnic Group during second wave

	M	Total (n=6074)θ	Latin Americans (n=464; 7.6%)θ	North Africans (n=19; 1.9%)θ	Sub-Saharan Africans (n=51; 0.8%)θ	Asians (n=34; 0.6%)θ	Europeans (n=5407; 89.0) θ	Global *p*	LA vs. E *p*	NA vs. E *p*	SS vs E *p*	Asian vs. E *p*
**Main outcomes, n (%)**												
**In-hospital mortality**	0	1117 (18.4)	23 (5.0)	19 (16.1)	1 (2.0)	4 (11.8)	1070 (19.8)	<0.001	<0.001	0.319	0.001	0.241
**Intensive care unit admission**	0	609 (10.0)	69 (14.9)	27 (22.9)	4 (7.8)	6 (17.6)	503 (9.3)	<0.001	<0.001	<0.001	0.721	0.096
**Invasive mechanical ventilation**	92	1664 (7.3)	185 (10.1)	30 (10.8)	9 (7.3)	10 (9.1)	1430 (7.0)	<0.001	<0.001	0.014	0.880	0.384
**First wave**	12	372 (6.1)	37 (8.0)	16 (13.6)	1 (2.0)	3 (8.8)	315 (5.8)	0.002	0.059	<0.001	0.239	0.460
**Composite outcome**	7	1499 (24.7)	73 (15.8)	33 (2.8)	5 (9.8)	7 (20.6)	1381 (25.6)	<0.001	<0.001	0.555	0.010	0.507
**Other outcomes, n (%)**												
**Non-invasive mechanical ventilation**	15	391 (6.5)	29 (6.3)	17 (14.4)	2 (3.9)	4 (11.8)	339 (6.3)	0.005	0.954	<0.001	0.490	0.190
**High-flow oxygen therapy**	13	761 12.6)	78 (16.8)	78(16.8)	7 (13.7)	4 (11.8)	645 (12.0)	<0.001	0.002	<0.001	0.664	0.73
**Length of hospital stay, days, median (IQR)**	11	9 (6-18)	7 (5-11)	8 (5-15)	8 (6-11)	7 (4-18)	9 (6-18)	<0.001	<0.001	<0.001	<0.001	<0.001
**Length of hospital stay >10 days, n (%)**	11	2723 (45.4)	166 (35.9)	51 (44.0)	17 (34.0)	13 (38.2)	2476 (46,7)	<0.001	0.002	0.213	0.023	0.035

θ Second wave; M: Missing value; E: Europeans; LA: Latin Americans; NA: North Africans; SS: Sub-Saharan Africans
